# Automated Skin Lesion Classification on Ultrasound Images

**DOI:** 10.3390/diagnostics11071207

**Published:** 2021-07-03

**Authors:** Péter Marosán-Vilimszky, Klára Szalai, András Horváth, Domonkos Csabai, Krisztián Füzesi, Gergely Csány, Miklós Gyöngy

**Affiliations:** 1 Faculty of Information Technology and Bionics, Pázmány Péter Catholic University, Práter u. 50/A, 1083 Budapest, Hungary; horvath.andras@itk.ppke.hu (A.H.); gyongy.miklos@itk.ppke.hu (M.G.); 2 Dermus Kft., Sopron út 64, 1116 Budapest, Hungary; domonkos.csabai@dermusvision.com (D.C.); krisztian.fuzesi@dermusvision.com (K.F.); gergely.csany@dermusvision.com (G.C.); 3 Department of Dermatology, Venereology and Dermatooncology, Semmelweis University, Mária u. 41, 1085 Budapest, Hungary; klaraszalai@gmail.com

**Keywords:** skin ultrasound, computer vision, computer-aided diagnosis, skin lesion classification

## Abstract

The growing incidence of skin cancer makes computer-aided diagnosis tools for this group of diseases increasingly important. The use of ultrasound has the potential to complement information from optical dermoscopy. The current work presents a fully automatic classification framework utilizing fully-automated (FA) segmentation and compares it with classification using two semi-automated (SA) segmentation methods. Ultrasound recordings were taken from a total of 310 lesions (70 melanoma, 130 basal cell carcinoma and 110 benign nevi). A support vector machine (SVM) model was trained on 62 features, with ten-fold cross-validation. Six classification tasks were considered, namely all the possible permutations of one class versus one or two remaining classes. The receiver operating characteristic (*ROC*) area under the curve (*AUC*) as well as the accuracy (*ACC*) were measured. The best classification was obtained for the classification of nevi from cancerous lesions (melanoma, basal cell carcinoma), with *AUC*s of over 90% and *ACC*s of over 85% obtained with all segmentation methods. Previous works have either not implemented FA ultrasound-based skin cancer classification (making diagnosis more lengthy and operator-dependent), or are unclear in their classification results. Furthermore, the current work is the first to assess the effect of implementing FA instead of SA classification, with FA classification never degrading performance (in terms of *AUC* or *ACC*) by more than 5%.

## 1. Introduction

### 1.1. Motivation

Skin cancer is a disease that is causing a growing problem in the developed world. For instance, one in five Americans are expected to get skin cancer during their lifetime, with an estimated 5.8% rise in melanoma cases for 2021 and a 77% rise in the incidence of non-melanoma skin cancer between 1994 and 2014 [1]. While malignant melanoma (MM) is the most deadly form of skin cancer, thankfully it is about 20 times less common than other forms of skin cancer, with basal cell carcinoma (BCC) being the most common non-melanoma skin cancer [2]. Due to the relative shortage of dermatologists in the midst of increases in skin cancer incidence, the role of computer aided diagnostic approaches is gaining increasing prominence.

Deep neural network-based optical approaches using tens of thousands of clinical records, including dermoscopy images, have achieved an accuracy of around 94% on automated skin lesion classification [3,4]. Despite the high accuracy of optics-based melanoma detection, the addition of subsurface information from ultrasound imaging can further improve classification accuracy [5].

In the last few decades, there has been increased interest in the use of dermatologic ultrasound for skin lesion diagnosis. The appearance of different cancerous and noncancerous skin lesions on ultrasound images, as well as their quantitative acoustical parameters, have been extensively reported [6,7,8,9,10,11,12,13,14,15,16,17,18]. In the following two subsections, ultrasound-based lesion diagnostic methods in general are briefly presented, followed by a brief review of the use of the aforementioned skin-specific features for skin cancer diagnosis.

### 1.2. Overview of Ultrasound-Based Lesion Diagnostic Methods

There are several lesion types, the automated classification of which is commonly studied in ultrasound imaging. Some of these are: benign versus malignant breast lesion differentiation [19,20,21,22,23,24]; thyroid cancer detection [25,26,27,28] or liver disease classification [29,30]. Lesion diagnosis techniques belong either to the traditional Computer-Aided Diagnostic (CAD) class of methods, or to the relatively recent class of deep learning (DL) methods. Both groups are briefly considered in turn, with the reader referred to the following review articles for a more extensive overview [31,32,33,34,35].

Typically, the CAD system pipeline consists of four main steps, namely pre-processing, segmentation, feature extraction, and classification. Unlike DL methods, CAD methods reduce the large size of the data input to a relatively small number of explicitly defined features. In the context of ultrasound-imaged lesions, previous works [31,34] have shown that promising features are: texture features, such as Laws’ texture energy, local binary patterns, wavelet features, contrast of gray level values or gray level co-occurrence matrices [21,22,25,26,29,30]; morphological features, such as spiculation, depth-to-width ratio, elliptic-normalized circumference and skeleton, long axis-to-short axis ratio [20,21,22]; statistical-model-based features such as those based on the Nakagami or K-Distributions [19]; and finally, bioinspired or domain-knowledge-based descriptors, such as those describing shape, calcifications, posterior shadow and echo, or echo characteristics [23].

In contrast to traditional CAD methods, DL methods, which do not require predefined features, have experienced an upsurge in popularity in virtually all areas of image processing. Due to their considerable ability in approximating arbitrary functions, DL methods can potentially replace any number of the above steps in the traditional CAD pipeline, provided that a sufficient number of training data are made available. This requirement is usually met in the case of “grand challenges” using popular lesion types, where anyone can test their algorithm on publicly available datasets. Recent examples of the use of DL in ultrasound-based lesion diagnosis are to be found in the classification of thyroid nodules [27,28], breast lesion differentiation [24] and lung ultrasound for the detection of COVID-19-associated lesions [36].

Despite their understandable popularity, since DL methods are based on large interconnected neural networks, their explainability (and thus the predictability of failure cases) poses a challenge to their verification. Moreover, as hinted earlier, DL techniques require large datasets (typically on the order of 1000 recordings). Due to the current relative lack of large datasets for skin ultrasound, ultrasound-based skin lesion classification techniques mostly rely on traditional CAD systems. This is the topic of the next subsection.

### 1.3. Ultrasound-Based Differential Diagnosis of Benign and Malignant Skin Lesions

Regarding the topic of skin cancer, the reader is first directed to two reviews of skin cancer detection methods in general [37,38], followed by two reviews of ultrasound-based skin cancer diagnosis [39,40]. These reviews of ultrasound methods highlight a number of studies where a number of quantitative and semi-quantitative parameters—such as echogenicity, homogeneity, shape, margins and location of the lesions, as well as the posterior acoustic shadow and dermal echogenicity ratio—are shown to be promising features in differential diagnosis [41,42,43,44,45,46,47]. These works, however, do not aim to provide automated classification, as they require fully manual segmentation and examine the diagnostic potential of features rather than combining them into a classification framework. Therefore, lesion classification accuracy values are either missing, not detailed properly, or do not reach the desired level (60%+) [40].

A number of more recent studies have moved towards providing a skin cancer classification framework. Csabai et al. [48] and Andrékuté et al. [49] combined an semi-automated (SA) segmentation method with a fully-automatic feature extraction and classification method based on acoustical, textural and shape features. Csabai et al. [48] examined three kinds of lesion types, namely MMs, BCCs and benign nevi. Using a manually selected seeding region, an active contour model (ACM) was used to segment the lesion. Five shape features and seven first-order texture parameters were defined and their mean and standard deviation were input as features into a support vector machine (SVM) model. In terms of the area under the receiver operating characteristic (*ROC*) curve (*AUC*) metric, they reported a classification performance of 86% for the differentiation between nevi from cancerous lesions and 90% for BCC vs. nevi. Andrékuté et al. [49] used a somewhat different approach: following a manual selection of those A-lines that contained the lesion, the lesion boundaries were automatically calculated for each A-line independently (A-lines are one-dimensional sections of the B-mode image in the depth direction, the direction of propagation of the ultrasound pulse). From these A-lines, 29 features were extracted for binary classification between MMs and benign melanocytic skin tumors (MST). An *AUC* performance of 89.0±0.6% was obtained.

In another strand of research, Kia et al. [50] presented an automatic classification method for differentiating between healthy tissues, benign lesions, BCCs and melanomas. Although 98% sensitivity was attained, this was achieved at the cost of the specificity being only 5%, making the diagnostic value of the algorithm extremely limited. (Although, judging from the context in which the performance values were reported, it is possible the authors may have meant to write a specificity of 95%.) In addition, healthy skin without lesions was included in the testing set, making a comparison with other classification articles difficult, if they do not consider the differentiation of lesion-free skin necessary. A more recent work from the group is based on tissue frequency analysis [51]. It uses a 384-element-long feature vector from frequency space to train the above-mentioned neural network. The algorithm calculated the features using the whole sonograms without applying any kind of segmentation before it. Their work reached an accuracy of 95.9% using 220 malignant and 180 benign lesions for training, testing and evaluation; a very promising result. However, since the report did not specify the four classes of skin type for which differential diagnosis was performed, more reports from their work are needed before the method can be verified.

A final work worthy of mention is that of Tiwari et al. [5], where skin lesion classification is performed based on parameters collected and combined from a number of different imaging modalities, namely ultrasonograpy, dermatoscopy and spectrophotometry. Although the results are outstanding (with an *AUC* of 99.9%), purely ultrasound-based classification is unfortunately not proposed or evaluated in the work.

In the table below (Table 1), the performance of the most relevant ultrasound-based classification methods is presented. They have been selected from the literature included above on the basis of having clearly defined and documented performance measures differentiating between skin lesions only.

### 1.4. Aims of Current Work

The current work aims to present a framework for ultrasound-based skin cancer diagnosis that differentiates between three common skin lesion types: benign nevi, BCC and MM. In contrast to techniques that require some form of manual segmentation, the use of an automated segmentation method [52] makes skin cancer detection fully automatic, which, considering the time limits imposed on dermatological visits [53], would significantly improve the utility of the skin cancer detection method. Although many methods exist for optical-based automated skin cancer diagnosis [3,4], as mentioned earlier, ultrasound has the potential to improve on the accuracy of fully optical-based methods [5]. The aim of the current work, therefore, is to assess how an fully-automated (FA) skin lesion detection method compares with reference SA methods and to compare with relevant results in the literature.

## 2. Materials and Methods

In the current section, the data processing pipeline is described from the point of data collection to data processing using feature extraction, classification and, finally, measures to evaluate classification performance. All the code used in the work is available to download on GitHub (Available online: https://github.com/marosanp/skin-lesion-us, accessed on 20 June 2021).

### 2.1. Ultrasound Data Collection

Data were collected at the Department of Dermatology, Venereology and Dermatooncology, Semmelweis University, Budapest, Hungary, as part of an ethically approved study. Informed consent was obtained from the participating patients for the anonymised use of the data for research and publication [52].

**Figure 1:**
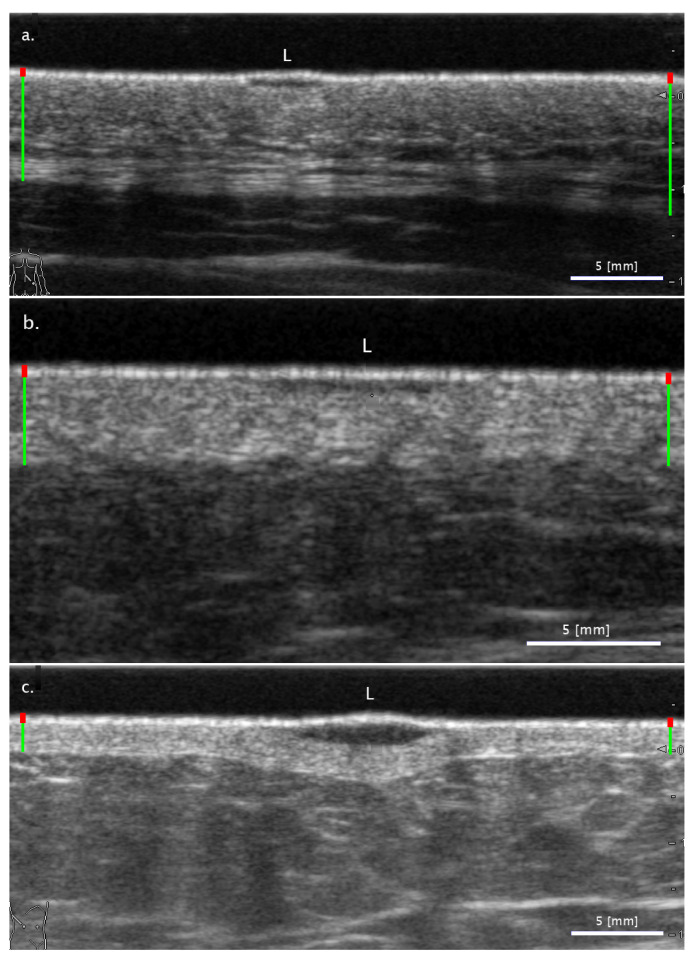
Representative ultrasound images of the three examined lesion types. (**a**) Nevus; (**b**) BCC; (**c**) MM. The primary layers of the skin are marked by the red (epidermis) and green (dermis) marks on the sides of the images. The lesions are marked by a white letter ‘L’ placed above them on the images.

The source of the examined dataset was a commercial high-frequency ultrasound imager (HI VISION Preirus with 5–18 MHz EUP-L75 transducer connected to Hitachi Preirus, Hitachi, Tokyo, Japan). The current study involved N = 310 B-mode ultrasound images, containing skin lesions with a thickness of 1–2 mm. Three different types of skin lesions were distinguished, including 110 benign nevi, 130 BCCs and 70 recordings of MMs. Figure 1 illustrates a representative ultrasound image of each examined lesion type.

### 2.2. Segmentation

Three different segmentation techniques were implemented and compared in the current study: one was an FA algorithm to study the accuracy of ultrasound-based FA skin cancer detection, while the other two were SA algorithms used as a reference. Note that the same segmentation method was used for training and testing rather than selecting one as the ground truth during training.

The first technique performs FA lesion segmentation based on an initial seeding step and a growing step, described in detail in [52], and described briefly below. The seeding step begins with a pre-processing substep to make ultrasound images from different machines similar to each other. This is followed by layer extraction substeps (above skin, epidermis, and dermis), and a lesion detection substep within the dermis. Each layer extraction substep first performs an intensity-based clustering or multilevel thresholding method combined with prior geometric information to return an initial estimate of the layer region; then performs a refinement of the region estimate based on an ACM and morphological operations. The seeding step is concluded by a lesion detection substep that incorporates information from the layer extraction substeps with prior geometrical assumptions about the arrangement of the layers and lesions. The automated seeding is followed by a growing step, using ACMs to extract the final lesion mask. To the best of our knowledge, this is the only fully automatic segmentation algorithm for ultrasound images of skin cancer-suspicious lesions that works on images from multiple imaging systems [52].

Two SA segmentation algorithms were also used for comparison purposes. Both of them require manual seeding for lesion localization and execute an ACM-based growing step on the initial seed masks for final boundary delimitation. The first, freehand seeding method used a freehand drawing around the lesion borders (using the MATLAB command freehand). This freehand seeding method simulated a careful manual segmentation since it allows any errors to be corrected using an ACM method. The second seeding method generated the largest area rectangle (LAR) from the freehand drawing and used that as the seed. This is similar to someone choosing a rectangular seed, as found in other works [54,55], and preferred in practice to freehand seeding due to the higher selection speed involved. We also have to mention that freehand seeding adds a significant variance and impairs the reproducibility of the algorithm as was demonstrated in [52]. In the current implementation, the difference was that the LAR was derived from the freehand seeding itself to allow meaningful comparison between the two.

The above-defined three segmentation techniques were chosen based on the following considerations. Our primary motivation of the work is to compare FA classification with SA classification. To the best of our knowledge, our previous work [52] is the only FA seeding-based method that was applied on more than one type of skin ultrasound image, demonstrating its robustness; therefore, this was chosen as the FA segmentation algorithm. Considering reference SA segmentation methods, freehand-seeding-based SA segmentation was considered a good approximation to ground truth, combining human knowledge with the filling in of spatially fine details. Since the freehand method is highly time-consuming, the LAR-based SA segmentation is considered a good simulation of a rapid human input into the segmentation workflow [52]. Since the freehand method was considered to be the most reliable segmentation, this was treated as the ground truth segmentation. In particular, the success rate of the FA segmentation was defined as the proportion of lesions where the Dice coefficient between the FA and freehand SA exceeded 10%.

### 2.3. Feature Extraction

Using the segmentation described in the previous subsection, 93 features were extracted from the lesion, lesion boundary, and the area of the dermis under the lesion. Then, feature selection was made by examining the SVM-based weights on the training set. In this way, 62 features were selected from 93 examined (Table 2).

The 62 features were calculated using the following image regions:

- Lesion region: all the pixels inside the lesion mask;
- Dermis region: pixels of the region of the dermis being right under the lesion mask;
- Lesion boundary: a lane of pixels being located within a fixed distance from the lesion mask boundary.

The features can be grouped into first-order textural, second-order textural, and shape features. First-order textural features express information about the distribution of individual pixel intensity values, while second-order textural features express spatial correlation between pixel intensities [56], and, in the current work, are based on the gray level co-occurrence matrix (GLCM) [57,58,59]. Lastly, some shape-based features were also extracted. All three groups of features are presented below.

#### 2.3.1. First-Order Textural Features

First-order textural features can be broadly categorized according to the properties or regions concerned, hence the subgroups; attenuation, lesion contrast, lesion boundary, and statistical.

Attenuation-based features, such as attenuation, contrast of attenuation and heterogeneity of attenuation, examine the lesion region and its shadowing in the dermis region right under the lesion mask. Contrast parameters, such as lesion- contrast-based heterogeneity and the mean lesion contrast, examine the contrast of each boundary line emanating radially from the inner edge of the lesion [48]. The above two subgroups, as well as the mean boundary belonging to the boundary subgroup, were adopted from Csabai et al. [48].

Lesion boundary region-based features, such as mean boundary, boundary heterogeneity, boundary contrast, boundary heterogeneity contrast, boundary-lesion contrast, dermis-lesion heterogeneity contrast and boundary-lesion heterogeneity contrast, are calculated based on the expressions presented in Table 2. Statistical features, such as skewness, kurtosis and entropy, were also selected.

#### 2.3.2. Second-Order Textural Features

For most of the second-order (GLCM) textural descriptors (contrast, correlation I. correlation II. dissimilarity, maximum probability, difference variance, difference entropy and information measure of correlation I.), the descriptors were calculated in both the vertical and horizontal directions, for both the lesion region and the dermis region of the images. For some of the second-order descriptors (energy, entropy, homogeneity I & II, and information measurement of correlation II) only the vertical GLCMs were calculated for both regions.

Further details and calculations of the above listed GLCM-based co-occurrence texture statistics are to be found in the work of Uppulari [59].

#### 2.3.3. Shape Features

Shape features are derived from parameters describing the shape of the lesion boundary. Some shape parameters, such as standard deviation of curvature and circularity, were extracted by Csabai et al. [48]. Further shape features, such as axis ratio, perimeter-area ratio and compactness (ratio of perimeter and the length of the major axis of the lesion mask), are also extracted.

The feature names with corresponding indices in the feature set (idx) are presented in Table 2, with references (to those taken from the literature) or with a short description (in the cases of newly introduced descriptors).

### 2.4. Classification

To aid comparison, the approach for the classification methodology (training and testing) closely resembled that of Andrékuté et al. [49]. Namely, an SVM-based classifier [60] was used, with ten-fold classification implemented in the following manner. First, ten separate groups were selected randomly, with the same ratios of lesion types. Then, each group in turn was selected as the test group, with the remaining nine groups merged into a training set. The classification performance was then averaged over the ten training instances, using accuracy (*ACC*) and *AUC* as performance metrics for the binary classification. In addition to binary classification (as implemented by Andrékuté et al. [49]), multiclass classification was also carried out in the current work and evaluated using *ACC*.

The following classes were distinguished for both binary and multiclass classification: ‘Nevus vs. others’, ‘MM vs. others’, ‘BCC vs. others’, ‘Nevus vs. BCC’, ‘Nevus vs. MM’, ‘BCC vs. MM’. In the cases of the binary classifications, ‘Nevus vs. BCC’, ‘Nevus vs. MM’ and ‘BCC vs. MM’ classifications were performed on datasets containing only lesions from the relevant two groups, while the corresponding multiclass classification results were obtained using datasets containing all three lesion types. ‘Nevus vs. others’, ‘MM vs. others’ and ‘BCC vs. others’ classifications were performed using datasets containing all three types of lesions in both binary and multiclass cases. Here, the binary classification training was conducted using two classes (a certain type of lesion versus all other lesions), while the multiclass classifications used three lesion type classes for training.

The parameter set (Algorithm 1) and the entire workflow (Algorithm 2) of the current work is presented as an algorithm in pseudo-code form at the end of the section, focusing on the details of the classification method. Details of the FA segmentation algorithm are presented in Marosán et al. [52], where figures of the procedure are also provided. Feature extraction is detailed in Table 2 and Section 2.3, Feature extraction.
**Algorithm 1:** Skin lesion classification algorithm—Part I. Variables and Methods.**Inputs**: *N*//where *N* is the number of ultrasound images collected $I_{1},I_{2},\ldots I_{N}$//$I_{j}$is the jth image collected (images are indexed by *j*) $L_{1},L_{2},\ldots L_{N}$//$L_{j}$is the lesion class of the jth image **Outputs**: $C_{1},C_{2},\ldots C_{N}$, where $C_{j}\in \left\{N,BCC,MM,O\right\}$//$C_{j}$is the predicted class label of the jth image (nevus, BCC, MM, other) **Parameters**: Seg//segmentation types: {FA, SA LAR, SA Freehand} Class//classification types: {‘Nevus vs. BCC’, ‘Nevus vs. MM’, ‘BCC vs. MM’, ‘Nevus vs. others’, ‘MM vs. others’, ‘BCC vs. others’} $mask_{L1},mask_{L2},\ldots mask_{LN}$//$mask_{Lj}$is the lesion mask of the jth image $mask_{D1},mask_{D2},\ldots mask_{DN}$//$mask_{Dj}$is the dermis mask of the jth image $F_{1},F_{2},\ldots F_{N}$//$F_{j}$is the feature vector of the jth image $F_{1}^{G},F_{2}^{G},\ldots F_{10}^{G}$//$F_{k}^{G}$is the kth group of feature vectors $C_{1}^{G},C_{2}^{G},\ldots C_{10}^{G}$//$C_{k}^{G}$is the kth group of label predictions of feature vectors **Methods**: $segment_{i}\left(\right)$//segmentation of lesion and dermis masks feature_extraction()//first and second order textural & shape feature extraction, based on dermis and lesion crops grouping()//selects randomly 10 groups, with the same ratios of lesion types labeling()//image labeling based on the following classification types: ‘Nevus vs. others’, ‘MM vs. others’, ‘BCC vs. others’, ‘Nevus vs. BCC’, ‘Nevus vs. MM’, ‘BCC vs. MM’ svm_train()//train SVM model, based on feature vector & label pair set svm_predict()//predict label for feature vector, using pre-defined SVM model evaluate()//compute *ACC* & *AUC* values based on pre-defined and predicted labels

### 2.5. Performance Metrics

The evaluation of classification methods was carried out using the performance metrics listed below. Numerous works have applied [48,49] the following metrics previously.

The sensitivity Sens calculates the proportion of positive cases that are correctly detected:

(1) $$Sens=\frac{TP}{TP+FN},$$

with TP, FN denoting the number of true positives and false negatives in the classification, respectively.
**Algorithm 2:** Skin lesion classification algorithm—Part II. Procedure.
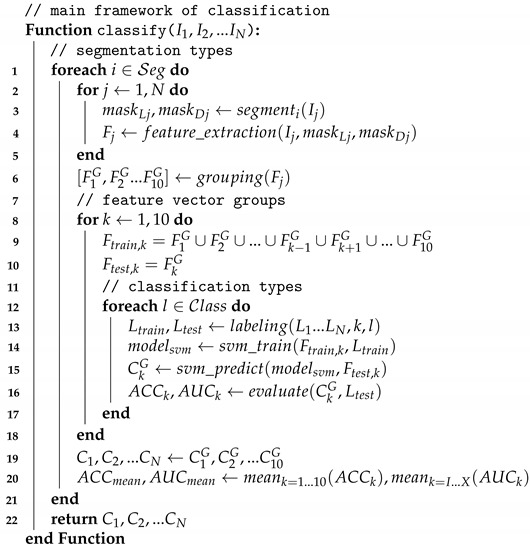


The specificity Spec calculates the proportion of negative cases that are correctly detected:

(2) $$Spec=\frac{TN}{TN+FP},$$

with TN, FP denoting the number of true negatives and false positives in the classification, respectively.

The accuracy ACC describes the classification accuracy, namely, the ratio of the number of correctly detected cases to the number of all examined cases

(3) $$ACC=\frac{TN+TP}{TN+TP+FN+FP}.$$

The receiver operating characteristic ROC curve is a graphical plot that displays how Sens varies with 1−Spec. Considering ROC as a function, its definition can be stated as

(4) $$Sens=ROC\left(1-Spec\right).$$

Finally, using the above definition for the ROC, the area under the *ROC* curve AUC can be defined as:

(5) $$AUC=\int_{0}^{1}ROC\left(x\right)dx,$$

where x=1−Spec.

## 3. Results and Discussion

### 3.1. Overview of Classification Performance

**Figure 2:**
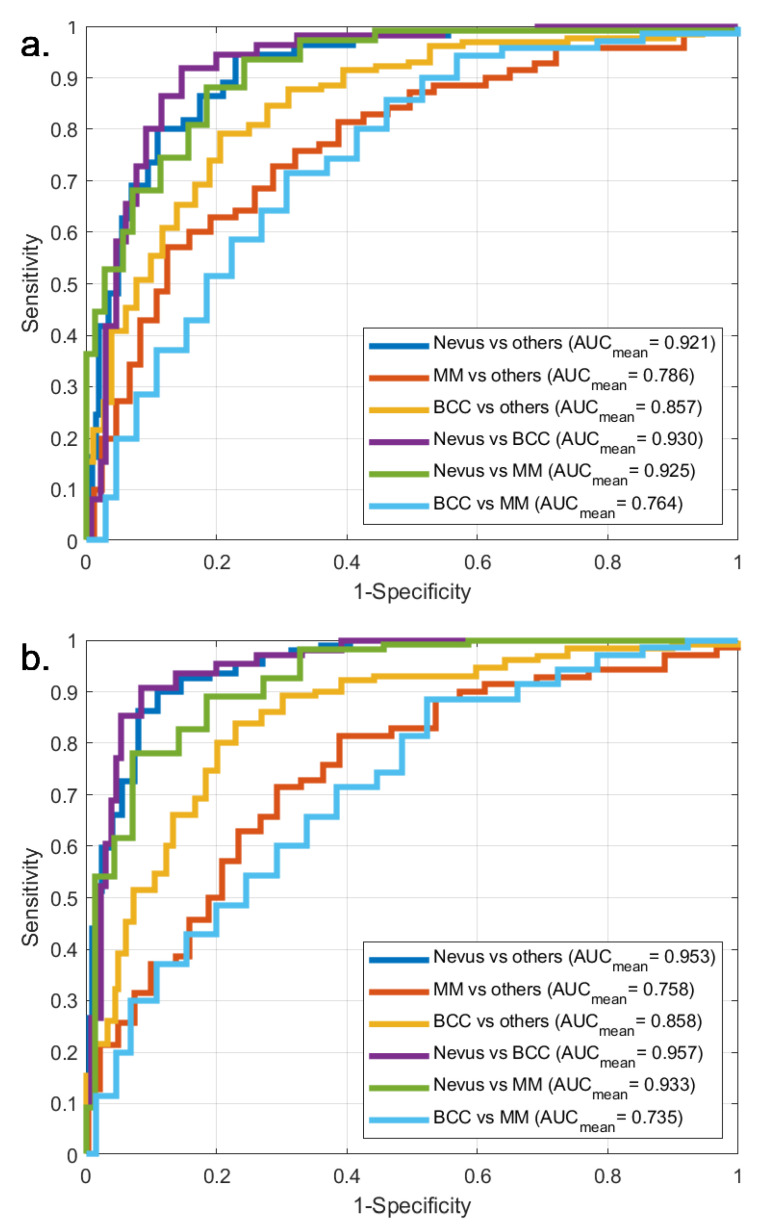

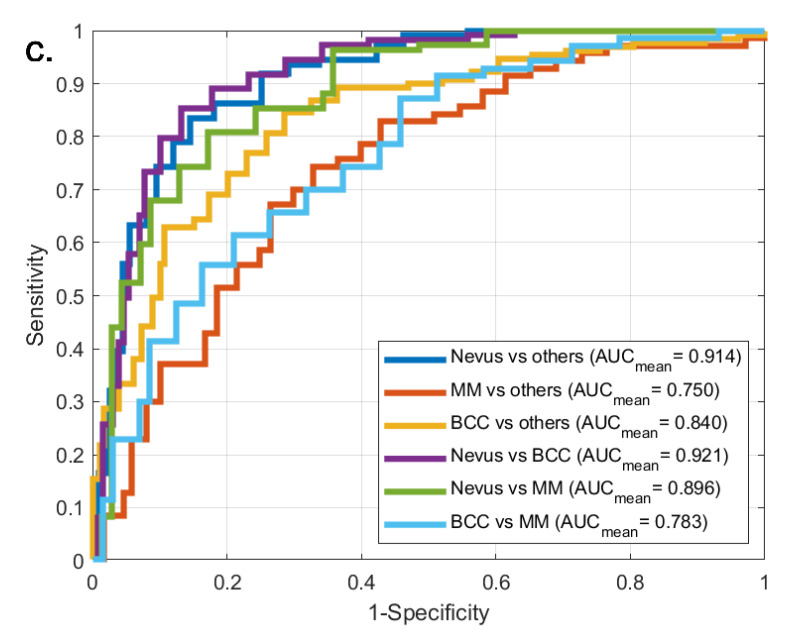
Receiver operating characteristic (*ROC*) curves using (**a**) freehand-seeding-based SA segmentation; (**b**) LAR-seeding-based SA segmentation; (**c**) FA segmentation and binary classification.

The *ROC* curves of the various binary classifications for all three segmentation methods are presented in Figure 2. From these curves, the *AUC* can be calculated, the values of which are summarized in Table 3.

In general, nevi are well distinguished from cancerous lesions: with one exception, the relevant classifications (‘Nevus vs. others’, ‘Nevus vs. BCC’, ‘Nevus vs. MM’) yield *AUC* values over 90% (highlighted in bold in the table).

Although the FA method generally fares worse than the SA methods, the performance is always within 5% of the best performing method.

The *ACC* values for both binary and multiclass classifications are summarized in Table 4. The table depicts a similar trend to the *AUC* results in Table 4: the nevi are generally well distinguished from cancerous lesions, with most such classifications reaching accuracies above 80% (highlighted in bold in the table). The notable exception is the multiclass classification of ’Nevus vs. MM’, which could be, in part, due to the relatively low number (N = 70) of melanoma recordings.

It should also be noted that multiclass classifications generally show a worse performance than binary classifications. This is arguably because multiclass classification forces the training classes to be smaller, outweighing the advantage that arises from being able to train on adequately distinct classes separately.

As before, the FA method generally shows a poorer performance; however, with one exception (‘BCC vs. others’, multiclass), the difference from the best method is never worse than 5%.

The current results also compare favourably to the results of Andrékuté et al. [49], where nevi were distinguished from melanoma with an accuracy of 82.4%: with the SA methods, the binary classification achieved an accuracy of 85.0%, while the FA method also achieved a comparable 81.1%.

For a comparison with other relevant works (all using SA segmentation), please see Table 1. Although the comparison is more difficult with other works, since they mostly set the sensitivity to 100% and then observe specificity, the current work nevertheless seems to fare well. For example, for those works where *AUC* values are provided, the current work is always superior in its corresponding *AUC* values. The specificity at 100% sensitivity shows more variable results in the current work.

Overall, the classification of nevi from cancerous lesions, and in particular from both cancerous lesion types (‘Nevus vs. others’), provides the best classification performance. Using multiclass classification, the FA method can distinguish nevi from cancerous lesions with an accuracy of over 85%.

### 3.2. Comparison of FA and SA Classification Performance with Representative Images

The reason behind a difference in classification performance can be a direct result of differences in segmentation during the classification phase; or it can be an indirect result of training on differently segmented images. For the FA method, 83.5% of the lesions were detected correctly, making its classification performance being close to that of the other methods somewhat surprising. To try and offer putative explanations for differences between classification performances, a presentation of images (with overlaid segmentation contours) that led to different classifications could be informative. In order to be consistent, the classification discussed will be the best performing one, namely ‘Nevus vs. others’. Before proceeding to discuss the images, it should be noted for context that, for this classification task, all three methods were jointly successful in 73.6% of the images, all three failed together in 5.2% of the cases, while there were differing classifications in 21.3% of the cases. In the following subsections, those cases where differing classifications are given are considered.

#### 3.2.1. Cases When FA Fails While SA Methods Perform Correctly

**Figure 3:**
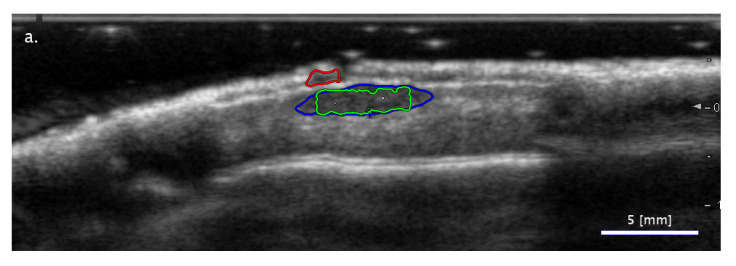

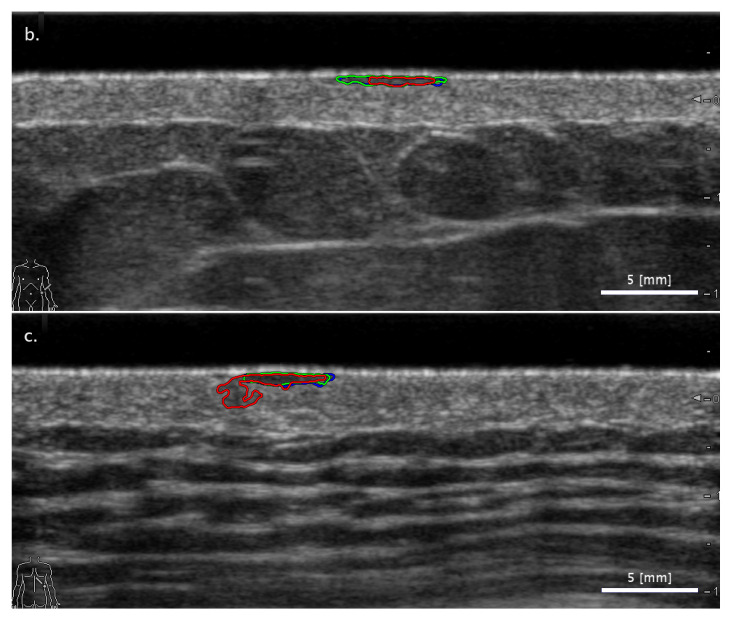
Examples of images with segmentation results leading to a correct ‘Nevus vs. others’ type binary classification in the cases of both SA methods (freehand: blue mark; LAR: green mark) while failing the classification results for the FA method (red mark). Three typical cases are presented: (**a**) an image region being totally separated from that of the lesion misleading the FA segmentation; (**b**) FA segmentation detecting the lesion only partially; (**c**) FA segmentation including misleading image areas nearby the lesion as part of the lesion segmentation.

In most cases of differing classifications, the SA methods succeeded while the FA method failed. Figure 3 presents examples for the three trends observed when analyzing this subset. In some cases, the FA segmentation method detected an image region fully outside the real lesion. As can be seen in Figure 3a, there are cases in which the image structure can be misleading when searching for the lesion location. In other cases, the FA segmentation did not detect parts of the lesion (Figure 3b) or included additional areas that were not part of the lesion (Figure 3c). In the latter case, the example shows a shadowing effect in the dermis region next to the lesion, which potentially misled the FA segmentation method.

#### 3.2.2. Cases When the Two SA Methods Return Different Classifications

**Figure 4:**
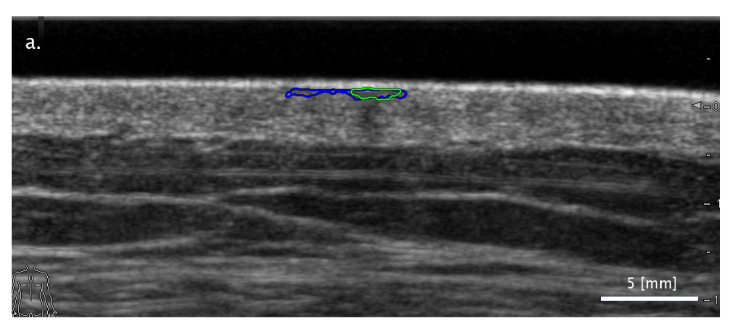

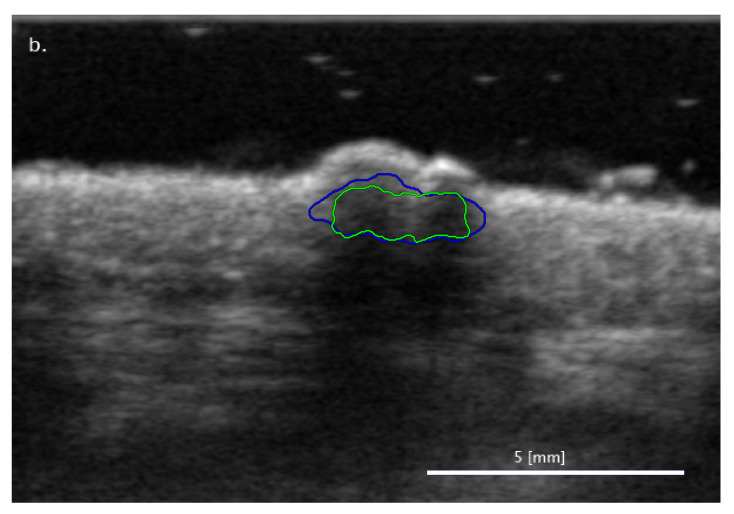
Examples of images with segmentation results leading to a different ‘Nevus vs. others’ type binary classification in the case of the SA methods (freehand: blue mark; LAR: green mark). Two typical cases are presented: (**a**) freehand segmentation leads to correct classification, but LAR-based segmentation detects the lesion only partially so its classification fails; (**b**) SA freehand segmentation fails because of arbitrary boundary selection, but the ACM model, used by SA LAR, corrects the mistake.

The SA segmentation methods, based on freehand and LAR segmentation, produced similar results generally; however, in certain cases, they led to different lesion classification results, such as in the cases presented in Figure 4. In the minority (29%) of such cases (2.5% of all images), the LAR-based method was the one failing the classifications. These were due to the shape of the lesion being such that the LAR segmentation could cover only a small portion of the lesion, which could not be expanded to cover the entire lesion even with the subsequent ACM method (Figure 4a).

In the majority (71%) of the cases discussed here (6.3% of all images), the freehand-based method led to failing classifications while the LAR produced correct results. These were presumably due to human error in the freehand segmentation, since the pixel boundaries of the freehand lesion segmentation are relatively arbitrary in contrast to the LAR-based method, where the ACM model finds the boundaries of the lesion with a higher precision (Figure 4b).

#### 3.2.3. Cases When the SA Methods Both Fail While the FA Method Performs Correctly

**Figure 5:**
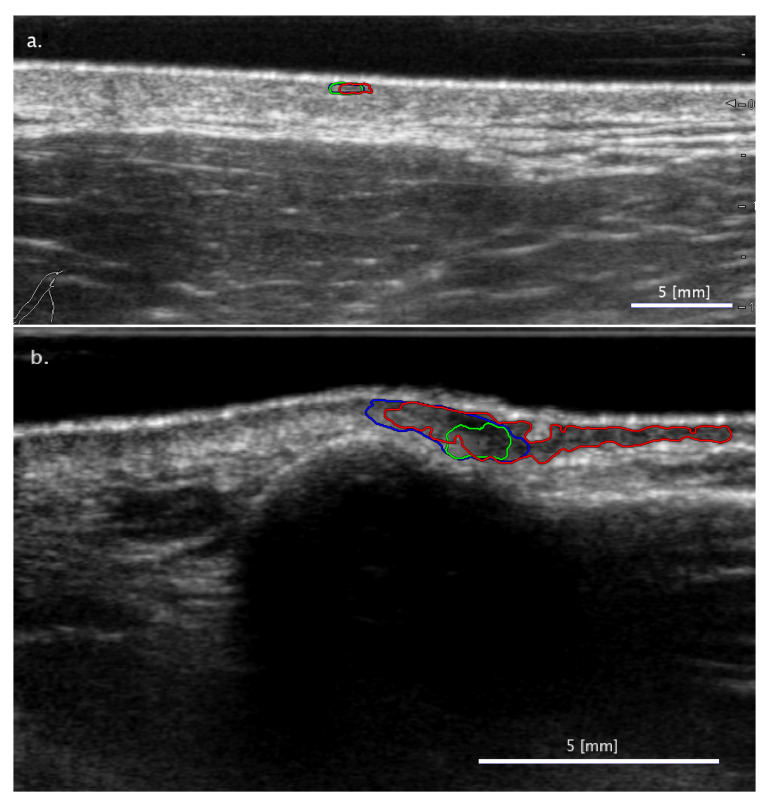

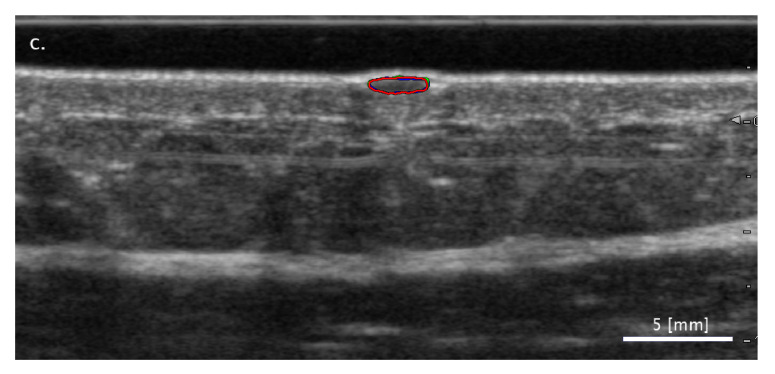
Examples of images with segmentation results leading to a correct ‘Nevus vs. others’ type binary classification in the case of the FA method (red mark) while failing for both SA methods (freehand: blue mark; LAR: green mark). Three notable cases are presented: (**a**) FA segmentation detected a similar but slightly shifted region from that detected by SA segmentation; (**b**) FA segmentation detected additional image regions as part of the lesion in comparison to the results of SA segmentations; (**c**) All three segmentation results matched closely, however slight differences in their borders led to different classification results.

In some cases, the ‘Nevus vs. others’ type classification was correct for the FA segmentation while failing for both SA segmentations. Figure 5 presents notable cases for such segmentations. Figure 5a shows a case in which the FA segmentation detected a similarly shaped and sized but slightly shifted region from that detected by the two SA segmentations. In some cases, such as the one presented in Figure 5b, the FA segmentation detected additional image regions as part of the lesion in comparison to the results of the SA segmentations, leading to a correct classification result.

In the case shown in Figure 5c, all three segmentation results matched closely; however, slight differences in their borders led to different classification results. This example emphasizes the significance of small details in automated ultrasound-image-based lesion classification performance.

### 3.3. Sensitivity of Classification to Changes in Lesion Segmentation

The last example in the previous subsection (Figure 5c) presented an interesting case, since it showed that slight changes in the border of the segmented lesion could lead to different classification results. Similar issues of classification sensitivity have been addressed elsewhere in the computer vision literature [61]; however, it is a challenging topic to address, partly due to the large search space involved in the sensitivity analysis. In the current work, the topic has been partially addressed in the following manner.

The ultrasound image on Figure 5 depicting a nevus was chosen as the target of the investigation, since this is where the phenomenon of changing classification due to a small change in lesion border was observed. Taking the freehand-based SA segmentation as the reference, the region was progressively grown/shrunk at various regions of the border (right edge, bottom edge, right and bottom, entire border), with the classification noted at each step.

**Figure 6:**
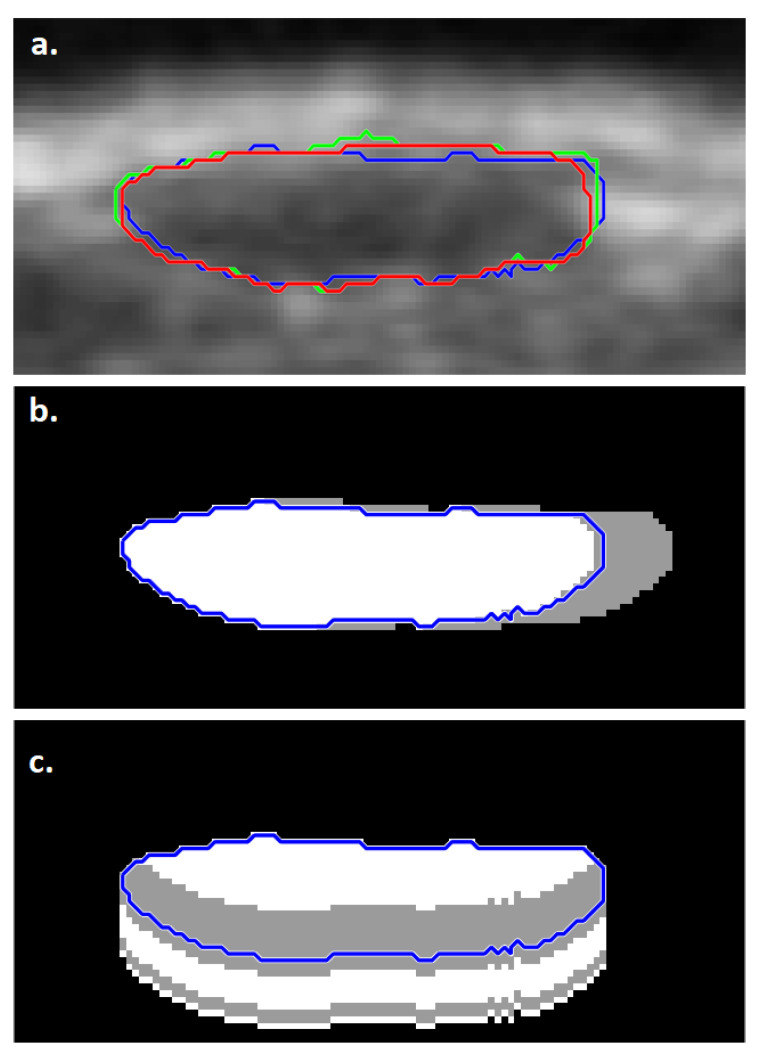

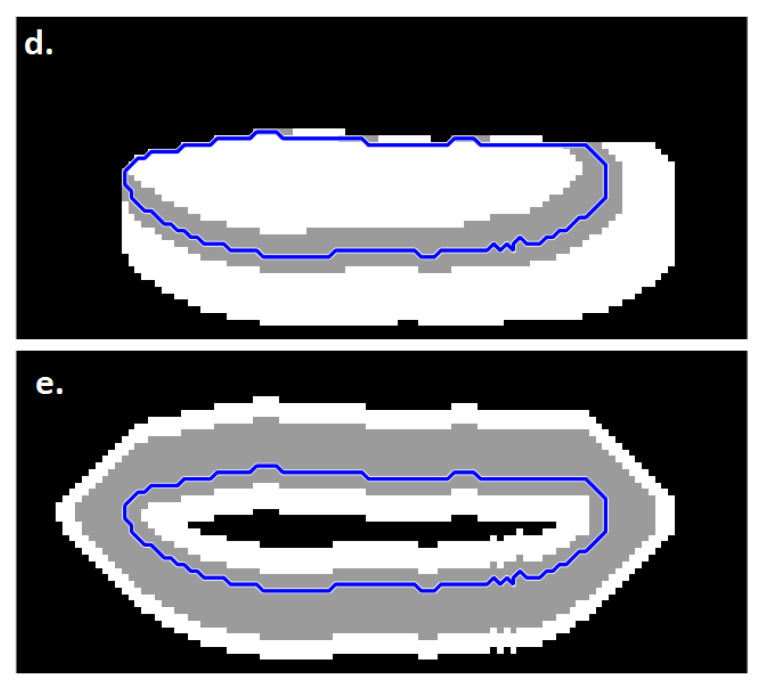
Sensitivity analysis of skin lesion classification procedure. (**a**) Zoomed ultrasound image from Figure 5c, with segmentations denoted using the previously used color scheme (freehand SA, LAR SA, FA). Taking the freehand-based SA lesion border as a reference, the border is shrunk/grown along various regions of the border ((**b**): right; (**c**): bottom; (**d**): right and bottom; (**e**): entire region). The classification result is drawn on the corresponding border change, with gray signifying incorrect classification, and white correct classification.

Figure 6 shows the results. Figure 6b shows that if the segmented region is shrunk only at the right edge, then a modest shrinkage of only 2 pixels’ width is able to change the classification from wrong to correct. This is consistent with the behaviour previously noted in Figure 5c, where the correct FA classification has a segmentation that is slightly shrunk at the right edge compared to the other two (Figure 6a). Interestingly, where other sides of the segmented regions are also modified, it becomes more difficult for the classification to be corrected. This could be because a hyperechoic patch on the right edge of the lesion perturbs the classification, and the segmentation is otherwise correct.

Although a more systematic analysis is beyond the scope of the current paper, the above preliminary analysis does show some insight into the relatively small yet significant perturbations in classification that can occur due to variabilities in segmentation.

### 3.4. Feature Performance

The aim of this section is to evaluate the features used in the classification framework by identifying those with the largest contribution to the classification performance. Since in the SVM classifier model the features do not act in isolation but are part of a non-linear system where features support each other, the performance of a feature was deemed to be better evaluated by measuring how much performance degraded when it was left out, rather than the performance it achieves on its own. Thus, the contribution of each of the 62 selected features was examined one-by-one as follows: one feature was removed and binary classification was performed using the remaining 61 features and using LAR-based segmentation. The *AUC* and *ACC* performance metrics were computed for all 62 cases for the six types of classification (similarly to Table 3 and Table 4 earlier when all features had been used). The top features—the absence of which caused the largest deterioration in classification performance—are shown in Table 5, with the worst feature also shown for reference. By removing one of the top features, the accuracy performance dropped by around 2 to 5%.

Table 5 shows several features that performed well on different classification cases, regarding both *AUC* and *ACC* performance measures. Those features that appear at least three times in the top four are in bold and are as follows (with feature indices in parentheses as listed in Section 2.3, Feature extraction): boundary heterogeneity (7); boundary contrast (8); skewness (13); entropy (15); axis ratio (18); compactness (20); and difference variance (26). Of these features, four are first-order textural features (7,8,13,15), two are shape-based features (18,20), and one is a second-order textural feature (26). When considering the possibility of applying the trained classifier to skin ultrasound images from other devices, the two shape-based features are arguably the most transposable; with the other features, some form of domain adaptation may be required [62]. In both the ‘Nevus vs. others’ and ‘Nevus vs. MM’ classification tasks, shape-based features were prominent, in agreement with the results of [49], where shape-based features provided the highest classification performance for distinguishing between nevi and melanoma.

### 3.5. Runtime Measurements

While the segmentation seeding algorithm was implemented in Python, the growing step, feature extraction, classification and evaluation were implemented in MATLAB R2018b (MathWorks, Inc., Natick, MA, USA). Table 6 details the computational cost of the proposed algorithm. Each ultrasound image had a size of 900×400 pixels and the computer used had an Intel Core i7-7500U CPU (2.70 GHz) processor and 16 GB RAM.

## 4. Conclusions

An automated framework for skin lesion classification was presented and an FA and two SA segmentation methods were compared. Ultrasound images of three types of lesions were used for the classifications: MMs, BCCs and benign nevi. Both binary and multiclass classifications were performed and evaluated, using six types of class differentiations, for all three segmentation methods, separately.

The best results were obtained generally for ‘Cancerous vs. Non-cancerous’ (‘Nevus vs. others’, ‘Nevus vs. BCC’, ‘Nevus vs. MM’) type binary classifications (>90% *AUC*). The two SA methods produced generally better results than the FA method, but with relatively slight differences (between 0.7–3.9%), such that the FA also provided an *AUC* at around 91% for the binary classification between nevi and the two cancerous lesion types. The achieved accuracies were similar to those obtained by Andrékuté et al. [49] when they differentiated between nevi and melanoma: 85.0% with the SA methods and 81.1% with the FA method, compared with 82.4%. The classification of nevi from cancerous lesions had even higher accuracies of above 85% even with the FA method. The result demonstrates the viability of FA skin cancer classification from ultrasound images.

Since features can be highly dependent on the type of ultrasound image they are applied to, it is worth noting that, in the case of the best performing classification task of distinguishing nevi from cancerous lesions, the top two performing features in terms of accuracy were shape-based features, since such features are more adaptable for different types of ultrasound images. Nevertheless, future work should focus on applying domain adaptation techniques to ensure the classification framework here presented can also be applied to skin ultrasound images produced by other devices.

## Figures and Tables

**Table 1:** Comparison of ultrasound-based skin lesion differential diagnosis methods focusing on benign and malignant skin lesion. In contrast to the current proposed method, none of the methods are fully automated since they do not employ fully-automated (FA) segmentation. NA stands for Not Available.

Lesions Compared	Features Used	Performance *AUC*/Sens./Spec.	Ref.
nevi vs. MM	acoustic shadowing, dermal echo ratio mean & std echogenicity, entry echo line	NA/100%/<30%	[44]
BCC vs. others	high frequency and Doppler ultrasound	NA/91%/14%	[45]
MM vs. others	echostructure, homogeneity, lesion margin Color Doppler, intralesional vessels	NA/100%/32% NA/100%/34%	[46] [46]
nevi vs. MM	surface & intra-lesional reflectance attenuation, param. relative uniformity	NA/100%/55%	[47]
nevi vs. cancerous	shape & texture features	86%/100%/19%	[48]
BCC vs. nevi	shape & texture features	90%/100%/45%	[48]
MM vs. MST	acoustical, textural & shape features	89%/85%/79%	[49]

**Table 2:** Feature set used for ultrasound-based skin lesion classification.

Feature	Idx	Reference/Description
**First-order textural features (Attenuation, Contrast, Boundary, Statistical features)**		
**Attenuation**		[48]
Attenuation	1	
Contrast of attenuation	2	
Heterogeneity of attenuation	3	
**Contrast**		[48]
Lesion contrast-based heterogeneity	4	
Mean lesion contrast	5	
**Boundary**		
Mean boundary	6	[48]
Boundary heterogeneity	7	std(LB)
Boundary contrast	8	avg(LB)/avg(L)
Boundary heterogeneity contrast	9	std(LB)/std(L)
Boundary-lesion contrast	10	[avg(LB)−avg(L)]/avg(LB)
Dermis-lesion heterogeneity contrast	11	[std(D)−std(L)]/std(D)
Boundary-lesion heterogeneity contrast	12	[std(LB)−std(L)]/std(LB)
**Statistical features**		
Skewness	13	
Kurtosis	14	
Entropy	15	
**Shape features**		
Standard deviation of curvature	16	[48]
Circularity	17	[48]
Axis ratio	18	Ma/ma
PA ratio	19	P/A
Compactness	20	P/Ma
**Second-order (GLCM) textural features**		[57,58,59]
Contrast	21, 29, 42, 50	
Correlation I.	22, 30, 43, 51	
Correlation II.	23, 31, 44, 52	
Dissimilarity	24, 32, 45, 53	
Energy	33, 54	
Entropy	34, 55	
Homogeneity I.	35, 56	
Homogeneity II.	36, 57	
Maximum probability	25, 37, 46, 58	
Difference variance	26, 38, 47, 59	
Difference entropy	27, 39, 48, 60	
Information measure of correlation I.	28, 40, 49, 61	
Information measure of correlation II.	41, 62	
**List of symbols**		
**avg—average**		
**std—standard deviation**		
**L—Lesion region (all the pixels inside the lesion mask)**		
**D—Dermis region (pixels of the region of the dermis being right under the lesion mask)**		
**LB—Lesion boundary (a lane of pixels being located within a fixed distance from the**		
**lesion mask edges)**		
**Ma—length of major axis of the lesion mask**		
**ma—length of minor axis of the lesion mask**		
**P—perimeter of lesion mask**		
**A—area of lesion mask**		

**Table 3:** Area under the curve (*AUC*) values of binary classification. Values over 90% are highlighted.

*AUC*	SA Segmentation	SA Segmentation	FA Segmentation
(Binary)	(Freehand)	(LAR)	
Nevus vs. others	**0.921**	**0.953**	**0.914**
MM vs. others	0.786	0.758	0.750
BCC vs. others	0.857	0.858	0.840
Nevus vs. BCC	**0.930**	**0.957**	**0.921**
Nevus vs. MM	**0.925**	**0.933**	0.896
BCC vs. MM	0.764	0.735	0.783

**Table 4:** Mean accuracy (*ACC*) values of binary/multiclass classification. Values over 80% are highlighted in **bold**.

Mean *ACC*	SA Segmentation	SA Segmentation	FA Segmentation
(Binary/Multiclass)	(Freehand)	(LAR)	
Nevus vs. others	**0.842**/**0.881**	**0.881**/**0.839**	**0.848**/**0.855**
MM vs. others	0.777/0.752	0.784/0.768	0.761/0.777
BCC vs. others	0.784/0.781	0.784/0.781	0.765/0.713
Nevus vs. BCC	**0.879**/**0.808**	**0.892**/0.792	**0.850**/0.750
Nevus vs. MM	**0.850**/0.661	**0.850**/0.667	**0.811**/0.689
BCC vs. MM	0.705/0.625	0.670/0.635	0.745/0.620

**Table 5:** Features performance. *AUC* (**a**) and *ACC* (**b**) measures of binary classifications using LAR-based segmentation are presented for the cases of leaving one feature out from the feature set of the classification framework. The results (*AUC* and *ACC*) are presented in ascending order, showing the omitted features in descending order regarding their contribution to the performance of the full framework. The features are represented by their index in the feature set of the framework. The top four rows for each of the six binary classification tasks are presented, together with the last row as a reference. The full list of the features (with feature names and corresponding indices) can be found in Section 2.3, Feature extraction. Features appearing at least three times in the top four are highlighted in **bold**.

a.											
**Nevus vs. Others**		**MM vs. Others**		**BCC vs. Others**		**Nevus vs. BCC**		**Nevus vs. MM**		**BCC vs. MM**	
idx	*AUC*	idx	*AUC*	idx	*AUC*	idx	*AUC*	idx	*AUC*	idx	*AUC*
**18**	0.910	**18**	0.738	**15**	0.850	**20**	0.927	**18**	0.866	11	0.738
**15**	0.913	**7**	0.777	4	0.850	49	0.927	**7**	0.905	4	0.747
**13**	0.918	34	0.780	9	0.851	2	0.928	**20**	0.908	**26**	0.749
7	0.918	32	0.780	32	0.854	10	0.928	9	0.909	17	0.752
...		...		...		...		...		...
55	0.929	11	0.794	14	0.864	55	0.937	4	0.930	18	0.771
**b.**											
**Nevus vs. Others**		**MM vs. Others**		**BCC vs. Others**		**Nevus vs. BCC**		**Nevus vs. MM**		**BCC vs. MM**	
idx	*ACC*	idx	*ACC*	idx	*ACC*	idx	*ACC*	idx	*ACC*	idx	*ACC*
**20**	0.826	1	0.771	**15**	0.768	6	0.875	**18**	0.789	**13**	0.680
**18**	0.832	**8**	0.771	34	0.774	**15**	0.875	**8**	0.828	**7**	0.690
41	0.832	11	0.771	6	0.777	21	0.879	**13**	0.833	21	0.690
43	0.832	**20**	0.771	**8**	0.777	**26**	0.879	**15**	0.839	**26**	0.690
...		...		...		...		...		...
9	0.868	26	0.784	39	0.790	8	0.896	19	0.861	5	0.725

**Table 6:** Runtime measurements of the proposed algorithm. For segmentation and feature extraction, the mean runtime over all images; for classification tasks, the mean runtime over all folds was calculated.

Method	Runtime Environment	Mean Runtime [s]
segmentation		
lesion detection	Python 3.7	2.209
border segmentation	MATLAB	2.414
feature extraction	MATLAB	0.377
binary classification		
training (lesion class vs. others)	MATLAB	3.129
training (class 1 vs. class 2)	MATLAB	2.043
prediction	MATLAB	0.002

## Abbreviations

The following abbreviations are used in this manuscript:

FA	fully-automated
SA	semi-automated
SVM	Support Vector Machine
*ROC*	receiver operating characteristic
*AUC*	area under the curve
*ACC*	classification accuracy
MM	malignant melanoma
BCC	basal cell carcinoma
CAD	Computer-Aided Diagnostic
DL	deep learning
MST	melanocytic skin tumor
NA	Not Available
LAR	largest area rectangle
ACM	active contour model
GLCM	gray level co-occurence matrix
